# COVID-Related Victimization, Racial Bias and Employment and Housing Disruption Increase Mental Health Risk Among U.S. Asian, Black and Latinx Adults

**DOI:** 10.3389/fpubh.2021.772236

**Published:** 2021-10-29

**Authors:** Celia B. Fisher, Xiangyu Tao, Tingting Liu, Salvatore Giorgi, Brenda Curtis

**Affiliations:** ^1^Department of Psychology, Fordham University, Bronx, NY, United States; ^2^Center for Ethics Education, Fordham University, Bronx, NY, United States; ^3^National Institutes of Health, National Institute on Drug Abuse, Baltimore, MD, United States; ^4^Positive Psychology Center, University of Pennsylvania, Philadelphia, PA, United States; ^5^Department of Computer and Information Science, University of Pennsylvania, Philadelphia, PA, United States

**Keywords:** COVID-19, mental health, victimization distress, racial bias, discrimination, housing, employment

## Abstract

**Background:** The mental health of racial/ethnic minorities in the U.S. has been disproportionately impacted by the COVID-19 pandemic. This study examined the extent to which disruptions in employment and housing, coronavirus-specific forms of victimization and racial bias independently and conjointly contributed to mental health risk among Asian, Black, and Latinx adults in the United States during the pandemic.

**Methods:** This study reports on data from 401 Asian, Black, and Latinx adults (age 18–72) who participated in a larger national online survey conducted from October 2020–June 2021, Measures included financial and health information, housing disruptions and distress in response to employment changes, coronavirus related victimization distress and perceived increases in racial bias, depression and anxiety.

**Results:** Asian participants had significantly higher levels of COVID-related victimization distress and perceived increases in racial bias than Black and Latinx. Young adults (<26 years old) were more vulnerable to depression, anxiety, and coronavirus victimization distress than older respondents. Having at least one COVID-related health risk, distress in response to changes in employment and housing disruptions, pandemic related victimization distress and perceived increases in racial bias were positively and significantly related to depression and anxiety. Structural equation modeling indicated COVID-related increases in racial bias mediated the effect of COVID-19 related victimization distress on depression and anxiety.

**Conclusions:** COVID-19 has created new pathways to mental health disparities among racial/ethnic minorities in the U.S. by exacerbating existing structural and societal inequities linked to race. Findings highlight the necessity of mental health services sensitive to specific challenges in employment and housing and social bias experienced by people of color during the current and future health crises.

## Introduction

The mental health of racial/ethnic minorities in the U.S. has been disproportionately impacted by the COVID-19 pandemic (1–4). Increases in depression and anxiety among people of color have been attributed in part to the exacerbation of existing health disparities and inequities in financial security (5, 6), especially through COVID-related employment and housing disruptions (7, 8). Discrimination against racial/ethnic minorities in the U.S. have also increased during the pandemic (9). A national representative survey by Pew Research Center (10) conducted in June 2020 reported that 31% Asian Americans, 21% Blacks, and 15% Latinx had been discriminated against due to their race/ethnicity. Moreover, the number of hate crimes in the U.S. during 2020 reported by FBI was the highest since 2008 (11). Direct and vicarious forms of racial/ethnic discrimination during the pandemic have been found to be positively associated with decreased mental health among racial/ethnic minority adults (12–15). A recent study involving a national sample of Asian, Black, Indigenous, and Latinx young adults ages 18–25 found the majority of respondents reported at least one instance of COVID-related victimization and when controlling for pre-existing COVID-19 medical risks and financial insecurity, perceptions that the coronavirus has increased racial bias across the country mediated the positive association between coronavirus victimization distress and depression and anxiety (16). These finding are consistent with past research finding long-term mental health consequences of contagious disease related discrimination among marginalized social groups in countries experiencing HIV, H1N1, and SARS COV-2 epidemics (17–20).

The social determinants of health and fundamental cause of population disparities theoretical frameworks, call for research that increases understanding of how the circumstances in which people work and live within the context of racially based stigma have detrimental consequences for mental health (21–23). Drawing on these frameworks, the current study sought to examine the extent to which disruptions in employment and housing and COVID-19 specific victimization and perceived increases in racial bias independently and conjointly contributed to depression and anxiety among Asian, Black, and Latinx adults in the United States. Consistent with these frameworks and based on prior research (16) we also tested whether perceptions regarding increases in COVID-related racial bias mediated the effect of coronavirus victimization distress on mental health risk.

## Methods

### Participants and Procedure

**Figure 1:**
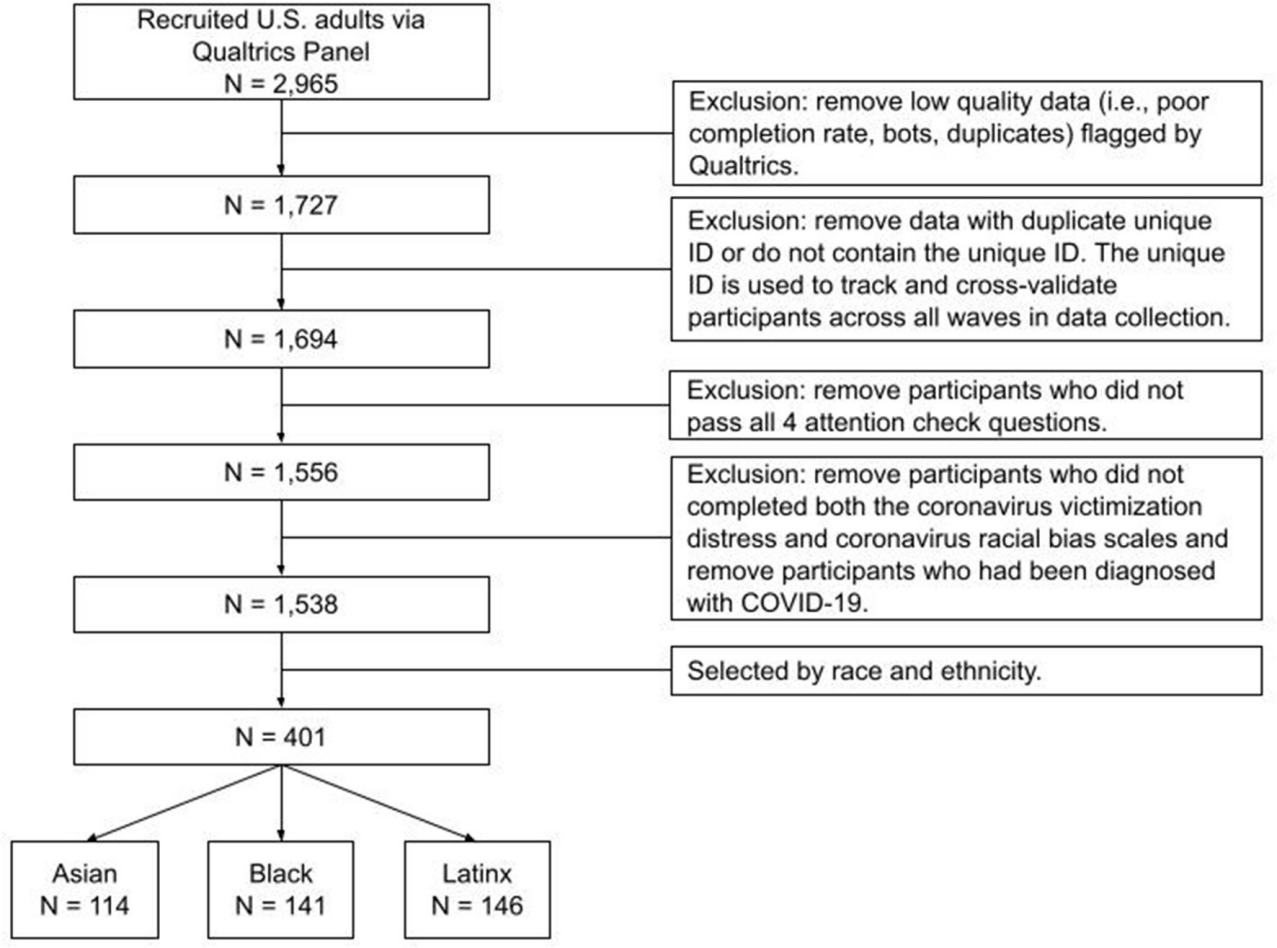
Flowchart of participant recruitment procedure. Participants who met the below three criteria would be invited: showed interests in the current study, use Facebook, age ≥ 18.

Data from the current study was part of a larger national survey recruited on social determinants of mental health and substance use among U.S. adults during the COVID-19 pandemic started from September 2020. Data used in the current study ranged from December 4, 2020 to June 4, 2021, with approximately 80% of the data collected during December 2020 to March 2021 (see Supplementary Tables 1, 2 for monthly recruitment break in the Supplement). During December 2020 to March 2021, COVID-19 situation is increasingly severe, according to the CDC (24), daily new cases increased from 213,919, reached the peak of 293,364 on Jan 8, and then decreased gradually to 73,074 by the end of March, which is still higher than most of the days in 2020. Similar trends were observed for daily deaths, which increased from 2,461, reached the peak of 4,169 in mid Jan, and then decreased gradually to less than 1,000. Mobility control of Americans is stable and the median user in the USA is traveling between 1 to 10 km (25). Participants were recruited online via Qualtrics Panel and need to be U.S. residents above 18 year-old and Facebook users to be included in the larger national survey. For data validity check, unique participant ID has been automatically created and assigned to each participant upon his/her initial participation in the larger national survey. This unique ID was used to track and cross-validate participants across waves of studies. In addition to IP address authentication (only IPs from the U.S.), four attention check questions were used throughout the survey. Though not present in the current study, the larger national survey collected participants' Facebook data, so the unique Facebook ID has also been used to validate participants across sections. In addition to the larger national survey recruitment criteria, for the present study, other inclusion criteria were as follows: self-reported Asian, Black, and Latinx race/ethnicity; completed the coronavirus victimization distress and coronavirus racial bias scales; had not been diagnosed with COVID-19; and passed data validity checks, see detailed recruitment procedure in Figure 1. Four-hundred-one participants met these criteria and entered the final data analysis. Participants received $70 upon completion of baseline questionnaires of demographics ($40) and the follow-up survey ($30).

### Measures

#### Demographics

Demographic information is presented in Table 1. Demographic variables included (a) race/ethnicity, gender, household income, and education level; (b) 7 medical conditions identified by the CDC as associated with risks of severe illness from COVID-19, including obesity, high blood pressure, lung disease, diabetes, heart or artery diseases, cancer, and HIV; (c) change in employment due to the pandemic and associated distress (response options ranged from 1 = “not at all troubled” to 5 = “extremely troubled”); and (d) housing disruption due to the pandemic as a proxy for financial insecurity (e.g. “I didn't pay the full amount of rent or mortgage”, “I was evicted from or asked to leave home”, “I didn't have a home”).

**Table 1:** Demographics and COVID-related employment and health variables among Asian, black, and Latinx adults.

	**Asian** ***N =* 114 (28.43%)**	**Black** ***N =* 141 (35.16%)**	**Latinx** ***N =* 146 (36.41%)**	**Total** ***N =* 401**	**Chi-square (df)**	***p***
	**frequency (%)**	**frequency (%)**	**frequency (%)**	**frequency (%)**		
Age						
18–25	37 (32.46)	16 (11.35)	31 (21.23)	84 (20.95)	17.97 (2)	**<0.001**
> 25	77 (67.54)	125 (88.65)	115 (78.77)	317 (79.05)		
Gender					2.20 (4)	0.70
Male	31 (27.19)	37 (26.24)	49 (33.56)	117 (29.18)		
Female	81 (71.05)	102 (72.34)	95 (65.07)	278 (69.33)		
Gender minority	2 (1.75)	2 (1.42)	2 (1.37)	6 (1.50)		
Household income					14.34 (4)	**0.006**
< $20,000	16 (14.04)	21 (14.89)	15 (10.27)	52 (12.97)		
$20,000–$50,000	21 (18.42)	52 (36.88)	37 (25.34)	110 (27.43)		
>50,000	77 (67.54)	68 (48.23)	94 (64.38)	239 (59.60)		
Education					36.66 (6)	**<0.001**
High school or technical/vocational school or less	4 (3.51)	20 (14.18)	12 (8.22)	36 (8.98)		
Some college	12 (10.53)	45 (31.91)	45 (30.82)	102 (25.44)		
Bachelor's degree	65 (57.02)	47 (33.33)	47 (32.19)	159 (39.65)		
Graduate degree	33 (28.95)	29 (20.57)	42 (28.77)	104 (25.94)		
COVID-19 health risk					
Obesity	9 (7.89)	39 (27.66)	34 (23.29)	82 (20.45)	16.28 (2)	**<0.001**
High blood pressure	9 (7.89)	35 (24.82)	18 (12.33)	62 (15.46)	15.54 (2)	**<0.001**
Lung disease	3 (2.63)	12 (8.51)	8 (5.48)	23 (5.74)	4.06 (2)	0.13
Diabetes	6 (5.26)	7 (4.96)	5 (3.42)	18 (4.49)	0.62 (2)	0.73
Heart or artery diseases	4 (3.51)	2 (1.42)	1 (0.68)	7 (1.75)	3.11 (2)	0.21
Cancer	2 (1.75)	2 (1.42)	1 (0.68)	5 (1.25)	0.65 (2)	0.73
HIV or AIDS	0 (0.00)	2 (1.42)	1 (0.68)	3 (0.75)	1.74 (2)	0.42
At least one of the above medical problems	21 (18.42)	66 (46.81)	50 (34.25)	137 (34.16)	22.59 (2)	**<0.001**
Employment changes due to pandemic	85 (74.56)	96 (68.09)	106 (72.60)	287 (71.57)	1.84 (2)	0.40
Housing changes due to pandemic	29 (25.44)	46 (32.62)	54 (36.99)	129 (32.17)	3.93 (2)	0.14
PHQ-9						
≥ Moderate depression	32 (28.07)	26 (18.44)	52 (35.62)	110 (27.43)	10.66 (2)	**0.004**
GAD-7						
≥ Moderate anxiety	33 (28.95)	39 (27.66)	45 (30.82)	117 (29.18)	0.35 (2)	0.84

*Mean Age for all participants = 35.54, SD = 11.01, range = 18–72 (M for Asian = 32.19, SD = 10.52, range = 19–72; M for Black = 38.04, SD = 10.87, range = 18–68; M for Latinx = 35.74, SD = 10.92, range = 18–69)*.

*Bold values represent statistically significant results*.

#### Coronavirus Victimization Distress Scale (CVDS)

Coronavirus victimization is defined as the experience of being singled out for cruel or unjust treatment because one is assumed to be a carrier of the COVID-19 virus. The CVDS (26) assessed 5 coronavirus related victimization experiences and associated distress. Items include being teased or bullied, physically threatened, mistreated, verbally taunted, called bad names, or cyberbullied because someone thought the respondent had the coronavirus. Responses were scored on a 5-point Likert-type scale (1 = “It never happened”, 2 = “It happened but did not upset me”; 3 = “It happened and upset me a little”; 4 = “It happened and upset me moderately”; 5 = “It happened and upset me quite a bit”). Prior research involving Asian, Black, Indigenous, and Latinx young adults (16) reported high inter-item reliability (Cronbach's alpha = 0.91) and the scale had good reliability for the current study (Cronbach's alpha = 0.92).

#### Coronavirus Racial Bias Scale (CRBS)

The 9-item CRBS (27) assessed participants' beliefs about how the coronavirus is negatively affecting societal attitudes toward one's race/ethnicity (e.g., “I believe the country has become more dangerous for people in my racial/ethnic group because of fear of the coronavirus”). Response options ranged from 1 (Strongly disagree) to 4 (Strongly agree). A prior study (16) conducted confirmatory factor analysis (CFA) on the CVDS and CRBS and found scale items loaded on distinct dimensions with one exception: there was a significantly correlated error between item 7 in the CRBS (i.e., “Due to the coronavirus I have been cyberbullied because of my race/ethnicity”) and item 5 in the CVDS (i.e., “I have been cyberbullied because someone thought I was infected with the coronavirus”); the revised scale had good reliability among racial/ethnic minority young adults (Cronbach's alpha = 0.87). Accordingly, item 7 was removed from the CRBS for the current study. The scale had good reliability for the current study (Cronbach's alpha = 0.82).

#### Patient Health Questionnaire (PHQ-9)

The PHQ-9 (28) assessed the frequency of past-month experiences with depressive symptoms. Sample items included “Little interest or pleasure in doing things” and “Feeling down, depressed, or hopeless”. Responses were scored on a 4-point scale, anchored by 0 (Not at all) and 3 (Nearly every day). Composite scale scores are computed by the sum of item responses. Prior research has shown internal consistency of the scale, with Cronbach's alpha ranged from 0.84 to 0.91 across racial/ethnic minority groups (16, 29, 30). The scale had good reliability for the current study, Cronbach's alpha = 0.91.

#### General Anxiety Disorder Screener (GAD-7)

The GAD-7 (31) assessed anxiety symptoms of participants during the past month. Sample items include “Being so restless that it is hard to sit still” and “Feeling afraid as if something awful might happen”. Response options are recorded on a 4-point Likert scale ranging from 0 (not at all) to 3 (nearly every day). Composite scale scores are computed by the sum of item responses. Internal consistency of the scale among racial/ethnic minority groups has been supported by prior research, with Cronbach's alpha ranged from 0.89 to 0.91 (16, 30). The scale had good reliability for the current study, Cronbach's alpha = 0.94.

### Data Analysis Plan

All analyses were conducted with R-4.0.1, and CFA and SEM analyses used *lavvan* package (32, 33).

#### Descriptive Statistics

Descriptive statistics were calculated for all demographic and COVID-19 related health and employment variables. This was followed by analyses of variance (ANOVA) and Chi-square tests to assess differences in these variables among the three racial/ethnic groups.

#### Confirmatory Factor Analysis

Confirmatory Factor Analysis (CFA) was then conducted to examine the structures of CVDS and CRBS, followed by descriptive statistics calculated for mental health indices and Chi-square tests, correlation and ANOVAs examining associations among demographic variables, CVDS, CRBS, and measures of depression and anxiety. Structural equation modeling (SEM) assessed the hypothesis that coronavirus racial bias exerted a mediating role in the association between coronavirus victimization distress and depression and anxiety. The goodness of fit indices included the comparative fit index (CFI), Tucker-Lewis Index (TLI), the root-mean-square error of approximation (RMSEA), and the Standardized Root Mean Square Residual (SRMR). A fit of > 0.95 for the CFI and TLI, < 0.06 for RMSEA, and < 0.08 for SRMR was considered to indicate adequate fit (34). To test the indirect effects for statistical significance, the bias-corrected bootstrapping approach was adopted as it is robust against the violation of normal distribution assumptions for both the sampling distribution and indirect effect (35). One thousand resamples were drawn to estimate the standard errors of the indirect effects and their 95% confidence intervals. Adequate power (=0.80) for the SEM analysis with degrees of freedom (df) = 25–60 has been achieved (36).

## Results

### Demographics and COVID-19 Related Employment and Health

Demographic data and Chi Square analyses by racial/ethnic group and the total sample are provided in Table 1. The sample included 401 participants (Mean age = 35.54, *SD* = 11.01, range = 18–72), who self-identified as, Asian (28.43%), Black (35.16%), and Latinx (36.41%). Across race/ethnicity, the majority identified as cisgender females (69.33%). Over half had completed college and reported a household income above $50,000. Thirty-four percent of participants had at least 1 COVID-19 health risk listed by the CDC (37). Asians were significantly younger than Black and Latinx groups, F(2, 398) = 9.30, *p* < 0.001. After adjusting for age, Asians reported higher levels of household income than Black, F(3, 400) = 8.30, *p* < 0.001. Both Asian and Latinx groups reported fewer medical conditions than Black, F(3, 400) = 15.08, *p* < 0.001, and Asians reported higher education levels than Black and Latinx, F(3, 400) = 11.40, *p* < 0.001.

#### Employment and Housing Disruption

As illustrated in Table 1, 71.57% of participants reported employment changes due to the pandemic, and 56.36% reported they were at least slightly troubled by employment changes (score > 2; *M* = 1.90, *SD* = 1.61); no racial/ethnic differences in employment disruption or distress were reported.

There were also no racial differences on housing changes after adjusted for age.

### Coronavirus Victimization Distress (CVD) and Coronavirus Racial Bias (CRB)

The CFA model included all five items on CVDS and four parcels constructed from the 8-item CRBS, which were created by randomly pairing two items together and calculating the mean. Results yielded a good model fit (CFI = 0.987, TLI = 0.981, RMSEA = 0.053, 90% CI [0.033, 0.072], and SRMR = 0.037) (See Supplementary Figure 1 in supplement materials). As indicated in sections 2.2.2 and 2.2.3 both scales had good reliability. Table 2 presents the means, standard deviations and range of scores on the CVDS and CRBS scales. In total 16.5% of participants reported at least one type of coronavirus victimization experience (27.2% Asian, 9.9% Black, and 14.4% Latinx). Chi-square analysis found that Asians were significantly more likely to report such experiences than Black and Latinx, χ^2^(2) = 14.38, *p* < 0.001 and young adults were significantly more likely to report at least one COVID-related victimization experience than adults over age 25 (68.18%), χ^2^(1) = 5.64, *p* = 0.018. As illustrated in Table 3 pandemic-related employment change distress was positively associated with both the CVDS and CRBS, and housing disruptions with the CRBS.

**Table 2:** Means, SD, and range for coronavirus victimization distress scale (CVDS), coronavirus racial bias scale (CRBS), PHQ-9 and GAD-7 for each racial/ethnic group.

	**Asian**	**Black**	**Latinx**	**Total**	**F (df1, df2)**	***p***
	**mean (S.D.)**	**mean (S.D.)**	**mean (S.D.)**	**mean (S.D.)**		
Coronavirus victimization distress (CVDS) ^a^	1.32 (0.78)	1.11 (0.48)	1.14 (0.54)	1.18 (0.60)	4.62 (2,398)	**0.01**
Coronavirus racial bias (CRBS) ^b^	2.48 (0.70)	2.28 (0.63)	2.14 (0.62)	2.29 (0.66)	9.12 (2,398)	**<0.001**
Depression (PHQ-9) ^c^	7.09 (6.41)	5.66 (5.46)	7.59 (6.57)	6.80 (6.20)	4.07 (2,398)	**0.018**
Anxiety (GAD-7) ^d^	7.02 (6.28)	5.92 (5.30)	7.47 (5.89)	6.80 (5.83)	2.65 (2,398)	0.07

a *Range = 1–5 for Asian and Latinx, 1–4.8 for Black*.

b *Range = 1–4 for Asian and Black, 1–3.78 for Latinx*.

c *Range = 1–27 for Asian and Latinx, 1–24 for Black*.

d *Range = 1–21 for Asian and Latinx, 1–20 for Black*.

*Bold values represent statistically significant results*.

**Table 3:** Pearson's correlations among coronavirus victimization distress (CVD), coronavirus racial bias (CRB), mental health indices and demographic and COVID-19 related employment and health variables.

	**1**	**2**	**3**	**4**	**5**	**6**	**7**	**8**	**9**	**10**
1. PHQ-9	1									
2. GAD-7	0.79^***^	1								
3. CVDS	0.22^***^	0.28^***^	1							
4. CRBS	0.22^***^	0.31^***^	0.29^***^	1						
5. COVID-19 health risk	0.15^**^	0.13^**^	−0.06	0.05	1					
6. Employment change distress	0.24^***^	0.30^***^	0.15^**^	0.17^**^	0.04	1				
7. Housing changes	0.25^***^	0.20^***^	0.08	0.14^**^	0.16^**^	0.36^***^	1			
8. Age	−0.17^**^	−0.14^**^	−0.08	−0.09	0.29^***^	0.07	0.009	1		
9. Household income	−0.19^**^	−0.16^**^	0.09	−0.09	−0.08	0.09	0.18^***^	0.18^***^	1	
10. Education level	−0.14^**^	−0.11^*^	0.02	−0.04	−0.04	−0.04	0.14^**^	0.15^**^	0.39^***^	1

* *p ≤ 0.05*

** *p ≤ 0.01*

*** *p ≤ 0.001*.

### Relationship of CVDS and CRBS and Demographic Variables With Mental Health Indices

Approximately 27% of participants met the criteria for moderate depression and 29% for anxiety as measured by PHQ-9 (≥ 10) and GAD-7 (≥ 10) (38) with no racial/ethnic differences. Young adults ages 18–25 were more likely to reach criteria for moderate depression (*N* = 32; 38.10%) compared to older adults (*N* = 78, 24.61%), χ^2^(1) = 6.07, *p* = 0.014). Latinx reported significantly higher levels of depression than Blacks (see Table 2). There were no gender differences on either mental health measure.

#### Correlations Among Variables

Before examining the SEM model, Pearson's and Spearman's correlation analyses were conducted to examine relationships between demographic variables, the CVDS and CRBS and the mental health indices (see Table 3). The CVDS, CRBS, having at least one COVID-19 health risk, employment change related distress, and housing disruption were all positively and significantly related to depression and anxiety as measure by the PHQ-9 GAD-7, respectively. Age, household income, and education level were negatively associated with depression and anxiety and age was positively associated with number of CDC health risks.

#### Structural Equation Modeling

**Figure 2:**
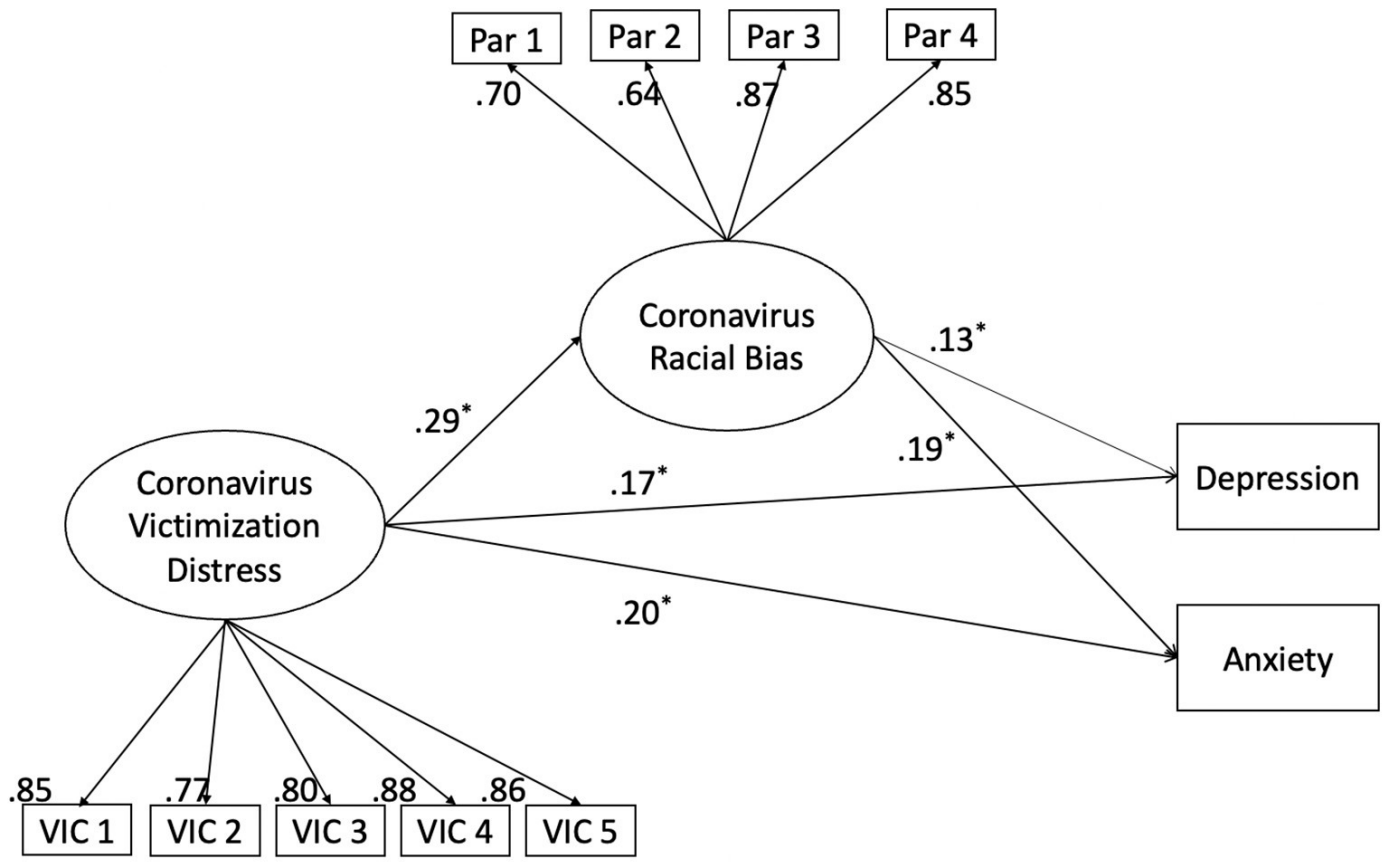
Standardized results for main structural equation model with bootstrapping approach testing the mediating effect of coronavirus racial bias on the association between coronavirus victimization distress and depression and anxiety. Covariates included race/ethnicity, COVID-19 health risks, employment change distress, housing changes, age, household income, and education level. *represents statistically significant results based on 95% Confidence Interval.

As described in the Analysis Plan and Figure 2, SEM was conducted to assess whether perceived coronavirus racial bias mediates the effect of coronavirus victimization distress on depression and anxiety. The model showed an adequate fit on all fit indices, including the CFI (0.973), TCL (0.963), RMSEA (0.044), 90% CI [0.033 0.055], and SRMR (0.034). Coronavirus victimization distress had significant direct effects on both depression and anxiety (Beta = 0.171, 95% CI [.238, 1.789], Beta = 0.199, 95% CI [0.483, 1.732], respectively) as well as indirect effects on mental health indices (Beta = 0.037, 95% CI [0.053, 0.434], Beta = 0.055, 95% CI [0.129, 0.518], respectively), indicating that coronavirus racial bias partially mediated the effect of victimization distress on depression and anxiety.

## Discussion

The COVID-19 pandemic has exacerbated existing health and social disparities among racial/ethnic populations in the U.S. accompanied by an increase in racial bias incidents (11). Understanding the effects on mental health of health, employment and housing disruptions and discrimination based on association with between the coronavirus and long-standing racial biases is essential to the development of adequate mental health services and prevention policies for Asian, Black and Latinx adults during the current and future pandemics (21, 39). Consistent with recent studies conducted during earlier stages of the pandemic, we found that having at least one COVID-19 health risk and experiencing coronavirus victimization was associated with higher mental health risk partially mediated by perceived coronavirus induced increases in racial bias (4, 16). This study also expands the work of prior findings by identifying distress caused by pandemic related housing and employment disruptions as not only a risk factor for depression and anxiety but as associated with increases in perceived COVID-related societal biases against Asian, Black and Latinx people in the U.S. The higher prevalence rate of coronavirus victimization experiences reported by our Asian participants can be understood within the context of the media focus on the origins of the virus in China fueling anti-Asian sentiment and reported increases in Asian bias incidents (16, 40, 41). The finding that Asian respondents were in general of higher household income and education and had less reported pre-existing COVID-19 health risks demonstrates the pernicious effect of racism on mental health irrespective of other historically documented protective factors (42).

### Limitations and Future Directions

The current study has limitations in participant recruitment procedure. The anonymous nature of online studies and national reach enables recruitment of geographically diverse participants. However, a limitation of all online surveys is the recruitment was limited to individuals who had previously registered for taking online surveys and thus may be different from those who are not registered with these recruitment sites. This study supported prior research indicating an association between pre-existing COVID-19 health risks and mental health. This association was especially strong for young adults in our sample although contrary to predictions based on social determinants of health, age was not associated with household income or education. Additional studies are needed to further explore factors that might contribute to these age differences. Though the data was not present in the current study, participants in the larger survey need to be Facebook users and share their Facebook data to researchers, this might further limit the current findings to people who use Facebook and future studies should involve a broader population. In addition, the current data was collected using a non-probability sampling method, additional studies are needed utilizing recruitment strategies that focus on more difficult to reach populations to reduce the potential sampling biases. Another potential limitation in recruitment is that our participants received monetary compensation upon the completion of the survey. Though the monetary incentives could affect survey completion, the compensation in the present study was divided into different waves of recruitment (baselines, follow-up, etc), and the current study used rigorous validation checks to ensure the quality of the data and to prevent potential conscious falsification, see details of strategies we used in recruitment procedure in Figure 1. We also have an unique ID that has been assigned to each participant and used to validate participants across waves of data collection. The current cross-sectional study illuminated significant associations among housing and employment disruptions, coronavirus victimization distress, perceived increases in pandemic related racial bias, and mental health among Asian, Black, and Latinx during the COVID-19 pandemic. However, the cross-sectional nature of the current study limits the causal interpretation of the results. Future longitudinal research will help identify the lasting mental health impact of coronavirus victimization distress and associated perceived systemic racism.

## Conclusion

This study demonstrates the importance of examining how pandemics can exacerbate existing systemic inequities experienced by members of socially marginalized racial/ethnic groups within the U.S. The mental health of Asian, Black and Latinx persons have long been associated with social discrimination and racial bias. Participant responses underscore how the COVID-19 pandemic has added to these mental health burdens through pre-existing health disparities, disruptions in employment and housing, and increased societal prejudices. COVID-19 has created new pathways to mental health disparities among adult members of these racial/ethnic groups. Findings highlight the necessity of mental health services sensitive to specific challenges in employment and housing and social bias experienced by people of color during the current and future health crises.

## Data Availability Statement

The datasets presented in this article are not readily available because assessments used in the current study could be provided upon request after being published. Due to privacy of participants, the data cannot be released in any format to any parties. Requests to access the datasets should be directed to BC, brenda.curtis@nih.gov.

## Ethics Statement

The studies involving human participants were reviewed and approved by University of Pennsylvania Institutional Review Board. Written informed consent for participation was not required for this study in accordance with the national legislation and the institutional requirements.

## Author Contributions

CF was a major contributor to study hypotheses, statistical analysis plan and interpretation, and writing the manuscript. XT conducted data analyses and interpretation and was a contributor in writing the manuscript. TL and SG contributed to survey design, data coding, interpretation of data analysis, and editing manuscript. BC was the major contributor responsible for study design, administration, data coding, interpretation of data analysis, and writing the manuscript. All authors read and approved the final manuscript.

## Funding

This study was funded by the Intramural Research Program (ZIA-DA000632-01) of the National Institutes of Health (NIH), National Institute on Drug Abuse (NIDA) and the Fordham University Center for Ethics Education. The authors report no financial relationships with commercial interests.

## Conflict of Interest

The authors declare that the research was conducted in the absence of any commercial or financial relationships that could be construed as a potential conflict of interest.

## Publisher's Note

All claims expressed in this article are solely those of the authors and do not necessarily represent those of their affiliated organizations, or those of the publisher, the editors and the reviewers. Any product that may be evaluated in this article, or claim that may be made by its manufacturer, is not guaranteed or endorsed by the publisher.
