# Supplementary material for: COVID-Related Victimization, Racial Bias and Employment and Housing Disruption Increase Mental Health Risk Among U.S. Asian, Black and Latinx Adults
Source: Front Public Health. 2021 Oct 29;9:772236. doi: 10.3389/fpubh.2021.772236 (PMC8585986; doi:10.3389/fpubh.2021.772236)

Supplement Materials

Table S1. Participant Response Rate.

|  | Total N |
| --- | --- |
| screener survey answered by | 20159 |
| Pass both validations (having FB & >=18) | 6359 |
| Finished baseline | 3752 |
| Finished 60 day | 2965 |

Table S2. Monthly Breakdown of Response Numbers for the 60-Day Survey.

| Month | 20-Dec | 21-Jan | 21-Feb | 21-Mar | 21-Apr | 21-May | 21-Jun |
| --- | --- | --- | --- | --- | --- | --- | --- |
| Number of Participants Finished the Survey | 473 | 598 | 295 | 668 | 241 | 320 | 370 |

Figure S1. Factor Loadings of CVDS and CRBS scales.


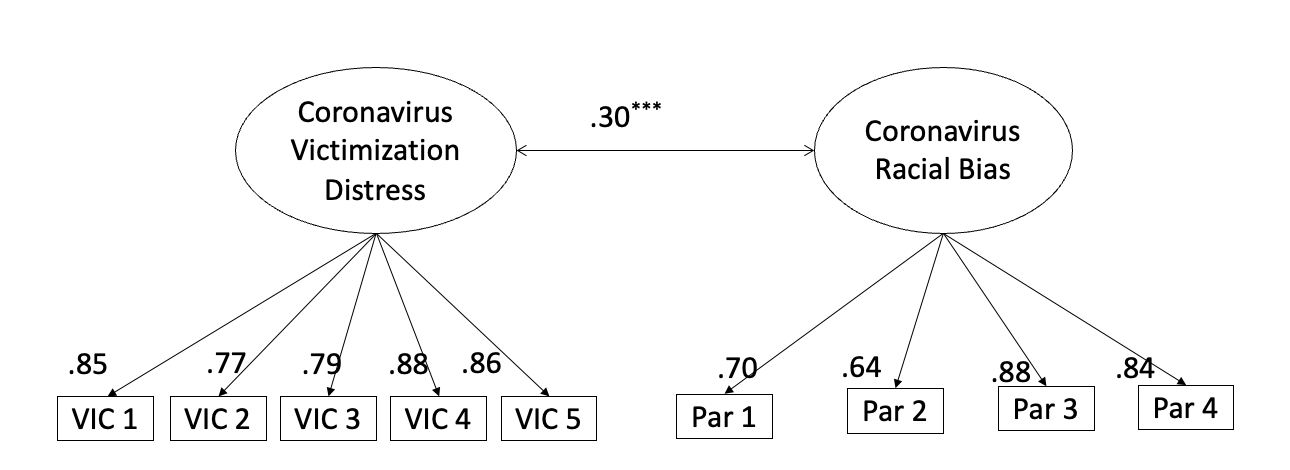

Supplement: Supplementary file 1 [file Table_1.docx]
